# Detection of a hydrogen sulfide-negative *Salmonella* Typhimurium from cattle feces in a cross-sectional study of cow–calf herds in the Southeastern United States

**DOI:** 10.3389/fvets.2025.1619880

**Published:** 2025-07-29

**Authors:** Kingsley E. Bentum, Emmanuel Kuufire, Rejoice Nyarku, Chase Woods, Khim Ale, Luis McKie, David McKenzie, Charlene R. Jackson, Abiodun Adesiyun, Tony Opoku-Agyemang, Temesgen Samuel, Gopal Reddy, Woubit Abebe

**Affiliations:** ^1^Center for Food Animal Health, Food Safety and Defense, College of Veterinary Medicine, Department of Pathobiology, Tuskegee University, Tuskegee, AL, United States; ^2^Department of Large Animal Clinical Science, College of Veterinary Medicine, Tuskegee University, Tuskegee, AL, United States; ^3^Poultry Microbiological Safety and Processing Research Unit, United States Department of Agriculture-Agriculture Research Service (USDA-ARS), U.S. National Poultry Research Center, Athens, GA, United States; ^4^Faculty of Medical Sciences, School of Veterinary Medicine, University of the West Indies, St. Augustine, Trinidad and Tobago; ^5^Department of Anatomy and Physiology, School of Veterinary Medicine, Kwame Nkrumah University of Science and Technology, Kumasi, Ghana

**Keywords:** *Salmonella*, antimicrobial resistance, fecal shedding, H_2_S-negative, mutation

## Abstract

**Introduction:**

Cattle are well-recognized reservoirs of *Salmonella*; however, reports of atypical hydrogen sulfide (H_2_S)-negative strains from bovine sources remain scarce. This cross-sectional study aimed to investigate the antimicrobial resistance profiles and epidemiology of *Salmonella* among rural cow-calf herds in Alabama, United States, with a particular focus on isolating emerging H_2_S-negative variants.

**Method:**

Between April and May 2024, a total of 311 fecal samples were collected from cattle across 18 farm operations in six counties. Samples were cultured for *Salmonella*, and recovered isolates were identified using whole genome sequencing and the *Salmonella In Silico* Typing Resource. H_2_S production was assessed using Xylose Lysine Deoxycholate agar, Lysine Iron Agar, and Triple Sugar Iron agar. Antimicrobial susceptibility testing was performed against 24 antimicrobial agents. A mixed-effects logistic regression model was used for statistical analysis.

**Results:**

Overall, 3.5% (11 out of 311) of animals from 27.8% (5 out of 18) of the farms tested positive for *Salmonella*. Fifteen isolates representing six serovars were identified: *Salmonella* Thompson (5 out of 15), *Salmonella* Hadar (4 out of 15), *Salmonella* Braenderup (3 out of 15), *Salmonella* Enteritidis (1 out of 15), *Salmonella* Bareilly (1 out of 15), and *Salmonella* Typhimurium (1 out of 15). Notably, the *tet(A)* gene conferring tetracycline resistance was detected exclusively in the *Salmonella* Hadar isolates. Diarrheic animals were significantly more likely to shed *Salmonella* in their feces (*p* value = 0.0192). Importantly, the *Salmonella* Typhimurium isolate was identified as an H_2_S-negative strain, carrying an A > C missense mutation in the *phsC* gene and a C > T synonymous mutation in the *cysI* gene.

**Conclusion:**

To our knowledge, this is the first report of an H_2_S-negative *Salmonella* Typhimurium isolate from cattle feces. These findings also reveal a notable prevalence of *Salmonella* shedding among an underexplored population of rural cow-calf herds in the southeastern United States. The potential public health implications of these findings merit further investigation.

## 1 Introduction

Pathogen surveillance and cross-sectional studies are critical epidemiological tools for investigating infectious agents and are essential for informing public health interventions (1–3). In the United States (U.S.), for instance, the National Animal Health Monitoring System (NAHMS) routinely conducts nationwide assessments to evaluate the health and management practices of various domestic animal populations (4). In fulfilling this role, NAHMS frequently employs surveillance methodologies, as demonstrated by its most recent 2021–2022 nationwide study of the U.S. swine industry (5). Within the cattle sector, NAHMS has successfully conducted four such national studies to date (6). Similarly, in China, surveillance efforts have led to the identification of 46 hydrogen sulfide (H_2_S)-negative *Salmonella* serovars from a range of human, animal, and environmental sources (7).

H_2_S-negative *Salmonella* serovars are considered “atypical” because “typical” *Salmonella* isolates produce H_2_S (8). In recent years, the global incidence of H_2_S-negative *Salmonella* isolates has increased (7), prompting increased calls for enhanced surveillance to support their detection (9, 10). Unfortunately, these atypical variants often go undetected in traditional *Salmonella* isolation procedures due to their deviation from the conventional phenotypic characteristics of the bacteria (11, 12). Specifically, H_2_S production on various culture media, manifested as “black colonies,” has long served as a key phenotypic marker for identifying *Salmonella* and distinguishing it from other members of the *Enterobacteriaceae* family (7, 8, 13, 14). However, the absence of this trait in H_2_S-negative strains increases the likelihood of false negatives during routine culture-based identification, unless detection methods that do not rely on H_2_S production, such as those reviewed by Yang et al. (15), are employed.

Furthermore, these atypical strains have been associated with clinical diseases and outbreaks (16, 17). In *Salmonella*, the *phs, cys*, and *asr* operons are key genomic regions involved in H_2_S production (17). The H_2_S-negative phenotype is primarily attributed to missense mutations in the *phs* operon, which plays a central role in the reduction of thiosulfate to H_2_S (12). Additional mutations, including those in the *cysJ* gene, have also been documented (17). In some isolates, mutations have been identified in other genes such as *tetR* (a putative transcriptional regulator of the TetR family), *moaC* (involved in molybdenum cofactor biosynthesis), and *sph* (streptomycin phosphotransferase) (12). Multiple studies across the globe have reported the isolation of these atypical *Salmonella* serovars from live animals and animal-derived products (8, 9, 14, 18, 19). However, aside from the identification of an H_2_S-negative *Salmonella* Cerro isolate from cattle and cattle farm environments in the U.S. (20), no other such isolates have been reported from bovine sources or their environments. This is particularly notable given that cattle are well-established reservoirs of *Salmonella* (21).

In the U. S., cattle production accounts for the largest share of total cash receipts from agricultural commodities, making it the most significant sector in U.S. agriculture (22). The two primary production systems in the U.S. beef industry are cow–calf operations and cattle-feeding operations (23). Compared to cow–calf operations, cattle-feeding operations are higher-risk ventures that economically favor large-scale establishments, whereas cow–calf systems are more adaptable for small-scale, part-time farmers (24). These cow–calf operations are widely distributed across the country (25), with the southeastern U.S. serving as home to approximately one-third of all cow–calf producers (26). This makes the region an ideal geographic area for conducting research on cow–calf populations.

Cow–calf operations focus on producing annual calf crops, which are typically weaned or sold at ~6 months of age, either as stockers (weaned calves up to 1 year of age) or as yearlings destined for cattle-feeding operations (27). These systems serve as an important source of income for many part-time farmers, who often rely on local extension services to manage their animals.

Despite the critical role cow–calf operations play in U.S. agriculture, research specifically focused on rural cow–calf systems and their contribution to the prevalence of key pathogens, such as *Salmonella*, remains limited. This gap is partly due to their underrepresentation in national surveillance studies, which often overlook rural operations because of geographic and demographic constraints.

To address this gap, we conducted a study during a scheduled animal extension activity in rural Alabama, as part of the Cooperative Extension Program (CEP) (28), that involved deworming and vaccinating local cow–calf herds. The objective of this study was to investigate the presence of H_2_S-negative *Salmonella* in cattle feces, as well as serovar diversity, antimicrobial resistance (AMR) profiles, and overall epidemiology of *Salmonella* among cow–calf farms in the southeastern U.S.

## 2 Materials and methods

### 2.1 Study area and sampling strategy

This cross-sectional study was conducted between April and May 2024 among small-scale cow–calf farms located across six counties in Alabama, in the southeastern U.S. A collaborative team of researchers and personnel from the Center for Food Animal Health, Food Safety, and Defense Laboratory at Tuskegee University, along with the Tuskegee University Large Animal Clinic, worked with county coordinators from the CEP to identify and select farms for participation.

The selected farms had herd sizes ranging from 1 to 50 cattle, with 70% of the herds consisting of at least 10 animals. County CEP coordinators assisted in enrolling eligible farms based on the following criteria: owner willingness to participate, the geographic location, ease of access, and the presence of at least two cattle in the herd. To minimize selection bias across farms, more than 50% of the animals in each selected herd were sampled.

Given an estimated total herd population of 1,500 cattle across the selected counties and the absence of prior prevalence studies in this population, the sample size was calculated using the formula provided by Thrusfield (29), and the following parameters were applied: a 95% confidence interval (*z*-score = 1.96); an expected animal-level prevalence of 28%, and a precision level of 0.05. The formula used was as follows:


$$\begin{array}{l} n=\frac{Z^{2}P\left(1-P\right)}{d^{2}}, \end{array}$$

where *n* represents the sample size, *Z* represents the *Z* statistic for a confidence level of 95%, *P* represents the expected prevalence, and *d* represents the estimated precision.

In summary, 311 fecal samples were collected from apparently healthy cattle across 18 different cow–calf operations during single, one-time farm visits, with no repeat sampling. The fecal grab technique was used for sample collection (30), with all samples obtained using sterile disposable gloves and deposited into sterile 50-ml container tubes. Sampling was conducted while the animals were restrained in a squeeze chute for routine deworming and vaccination. The collected samples were immediately placed in a cooler box on ice and transported to the laboratory within 3–4 h of collection for immediate bacterial culture and isolation. Sampled animals were classified by sex (male or female) and age group: calves (≤12 months), including both pre-weaned and weaned individuals, and adults (>12 months). Additionally, based on fecal consistency evaluated by the same personnel at each farm visit, animals were categorized as either non-diarrheic (producing firm or fairly consistent feces) or diarrheic (producing loose or watery feces). Of the 311 samples collected, 76 were from calves and 235 from adults; 273 were from females and 38 from males; and 76 were from diarrheic animals, while 235 were from non-diarrheic animals.

### 2.2 *Salmonella* culture and isolation

Screening of fecal samples for *Salmonella* was performed following the guidelines outlined in the U.S. Department of Agriculture, Food Safety and Inspection Service Microbiological Laboratory Guide (version 4.13) (31), with slight modifications. Briefly, to achieve a 1:9 sample-to-buffer ratio, 1 g of fecal matter was inoculated into 9 ml of Buffered Peptone Water (BPW) (Millipore Sigma, Danvers, MA, U.S.) in sterile 15-ml tubes and homogenized by vortexing for 2 min. The resulting fecal suspension was incubated in a shaker incubator at 37°C for 24 h.

Following this pre-enrichment step in BPW, selective enrichment for *Salmonella* was carried out. Specifically, 1.0 and 0.1 ml aliquots of the pre-enriched culture were transferred to 9 ml of Tetrathionate (TT) broth (Neogen, Lansing, MI, U.S.) and 10 ml of Rappaport-Vassiliadis (RV) broth (Millipore Sigma, Danvers, MA, U.S.), respectively. Both enrichment broths were incubated at 42°C ± 0.5°C for 24 h.

After incubation, ~20 μl from each TT and RV broth culture was streaked separately onto Xylose-Lysine-Desoxycholate (XLD) agar (Millipore Sigma, Danvers, MA, U.S.) and Hektoen Enteric (HE) agar (Neogen, Lansing, MI, U.S.). The plates were incubated at 37°C ± 2°C for 24–48 h. A minimum of three presumptive *Salmonella* colonies per plate, selected based on their characteristic colony morphology (32), were picked and subjected to biochemical confirmation.

Biochemical screening was performed by inoculating the colonies onto Triple Sugar Iron (TSI) agar slants (Neogen, Lansing, MI, U.S.) and Lysine Iron Agar (LIA) plates (Neogen, Lansing, MI, U.S.), followed by incubation at 37°C ± 2°C for 24 h. Isolates exhibiting typical biochemical profiles consistent with those of *Salmonella* (33) were further confirmed using Matrix-Assisted Laser Desorption Ionization–Time of Flight Mass Spectrometry (MALDI-TOF-MS; bioMérieux, Marcy-l'Étoile, France).

To confirm the H_2_S-negative phenotype, suspect isolates were plated on TSI, LIA, and XLD, using the *Salmonella* Typhimurium ATCC 13311 strain as a positive control. Finally, all confirmed isolates were preserved in Luria broth (Millipore Sigma, Danvers, MA, U.S.) supplemented with 30% glycerol and stored at −80°C until further analyses were performed.

### 2.3 DNA extraction from isolates, whole genome sequencing, and assembly

Genomic DNA was extracted from pure *Salmonella* isolates using the DNeasy UltraClean Microbial Kit (Qiagen, Hilden, Germany), following the manufacturer's instructions. The quality and quantity of the extracted DNA were assessed using a Bioanalyzer (Agilent Technologies, Santa Clara, CA, U.S.) and a Qubit 4 Fluorometer (Thermo Fisher Scientific, Waltham, MA, U.S.). Whole-genome sequencing was performed on the Illumina platform. Sample libraries were prepared using the Illumina DNA Preparation Kit and Nextera DNA CD Index Kits (Illumina, San Diego, CA, U.S.), followed by sequencing on the MiSeq platform using the 250 bp paired-end protocol.

Sequence quality was assessed using FastQC (http://www.bioinformatics.babraham.ac.uk/), and high-quality reads were assembled using the A5 pipeline (34). This pipeline includes five steps: read cleanup, read assembly, crude scaffolding, misassembly correction, and final scaffolding. The resulting contigs were used for downstream analyses.

### 2.4 Serovar identification, multi-locus sequence typing (MLST) of isolates and phylogeny

Serovar identification, including the determination of the somatic “O” and flagellar “H” antigens, was performed using the *Salmonella In silico* Typing Resource (SISTR) tool (version 1.1.2) (35). Sequence types (STs) were determined using the Achtman MLST scheme (36) via the PubMLST platform (37).

A phylogenetic tree based on single-nucleotide polymorphisms (SNPs) was constructed using the SNP Phylogeny tool of the Center for Food Safety and Applied Nutrition (CFSAN) pipeline via the Galaxy Platform (https://galaxy.sciensano.be/root). *Salmonella enterica* subsp. *enterica* serovar Typhimurium strain LT2 (NCBI accession number: AE006468.2) was used as the reference genome. The resulting phylogenetic tree, along with associated metadata, was visualized using the Interactive Tree of Life (iTOL) tool (38).

### 2.5 Antimicrobial susceptibility testing and resistance gene analysis

Antimicrobial susceptibility testing of the recovered isolates was conducted using the Sensititre™ National Antimicrobial Resistance Monitoring System (NARMS) Gram-negative GN4F plate (Thermo Scientific), which assesses susceptibility to 24 antibiotics. These antibiotics included amikacin, ampicillin, ampicillin/sulbactam, aztreonam, cefazolin, cefepime, ceftazidime, ceftriaxone, ciprofloxacin, doripenem, ertapenem, gentamicin, imipenem, levofloxacin, meropenem, minocycline, nitrofurantoin, piperacillin, tetracycline, ticarcillin/clavulanic acid, tigecycline, tobramycin, and trimethoprim/sulfamethoxazole.

Susceptibility interpretations followed the breakpoint criteria for *Enterobacteriaceae*, as outlined in the Clinical and Laboratory Standards Institute (CLSI) guidelines (39), except for nitrofurantoin and tigecycline, for which interpretation was based on previous studies (40–42).

To identify antimicrobial resistance genes, assembled genome sequences were screened against the Comprehensive Antimicrobial Resistance Database (CARD, version 4.0.0) (43) and ResFinder (version 4.3.3) (44), both accessed via ABRicate (version 1.0.1) (Seemann, Abricate, GitHub: https://github.com/tseemann/abricate) on 14 January 2025. The detection threshold for resistance genes was set at a minimum of 80% nucleotide identity and 80% sequence coverage.

### 2.6 Variation analysis on the hydrogen sulfide negative isolate

SNPs in the H_2_S-negative *Salmonella* Typhimurium isolate were analyzed using the variation analysis pipeline available on the Bacterial and Viral Bioinformatics Resource Center (BV-BRC) web-based platform (45), accessed on 19 February 2025. The sequence reads from the isolate were aligned to the reference genome of *Salmonella enterica* subsp. *enterica* serovar Typhimurium strain LT2 (NCBI Accession: AE006468.2) using the BWA-MEM algorithm (46). SNPs were then identified using FreeBayes (47), and the resulting variants, along with their predicted genetic effects, were evaluated using SNP-effect (48). Special focus was given to SNPs located in the *phs, cysJIH*, and *asr* operons, which are key genomic regions associated with H_2_S production in *Salmonella*.

### 2.7 Statistical analysis

A mixed-effects logistic regression analysis was performed using the generalized linear mixed model (GLMM) framework via the (lme4) package in R software (version 4.5.0) (49, 50). Farm groups of the animals were treated as a random effect, while age, sex, and diarrhea status were included as fixed effects. A *p-*value of < 0.05 was deemed statistically significant.

## 3 Results

### 3.1 Shedding of *Salmonella* among cattle from various farms

To maintain anonymity, the 18 farm-herds included in this study were labeled alphabetically from A to R (Table 1). At the herd level, *Salmonella* was isolated from 27.8% (five out of 18) of the farms visited. At the individual animal level, 3.5% (11 out of 311) of all cattle sampled tested positive for *Salmonella* (Table 1).

**Table 1:** Shedding of *Salmonella* among sampled animals.

**Farm**	**Total number of cattle on each farm**	**Animals sampled**	**Farm-level *Salmonella* detection**	**No. of positive animals (%)**
A	30	27	0	0 (0)
B	12	8	0	0 (0)
C	15	12	1	1 (8.3)
D	50	28	1	4 (14.3)
E	37	37	0	0 (0)
F	28	23	1	3 (13.0)
G	33	23	0	0 (0)
H	5	5	0	0 (0)
I	25	21	0	0 (0)
J	8	7	0	0 (0)
K	27	27	0	0 (0)
L	14	14	0	0 (0)
M	18	18	0	0 (0)
N	14	14	0	0 (0)
O	23	19	0	0 (0)
P	7	7	0	0 (0)
Q	7	7	1	1 (14.3)
R	14	14	1	2 (14.3)
***Total***	**367**	**311**	**5 (27.8)**	**11 (3.5)**

For farm-level Salmonella detection, 1 denotes “detected” and 0 denotes “not detected.”

For isolate identification, each isolate was labeled with the prefix “S” (for sample), followed by the isolate number and the suffix “_A” or “_B” to distinguish multiple isolates recovered from the same sample (Table 2). From the 11 *Salmonella-*positive animals, a total of 15 isolates were recovered, representing six distinct *Salmonella* serovars with their corresponding sequence types (STs). These isolates included the following: five *Salmonella* Thompson isolates (ST26), four *Salmonella* Hadar isolates (ST33), three *Salmonella* Braenderup isolates (ST22), and one isolate each of *Salmonella* Bareilly (ST464), *Salmonella* Enteritidis (ST11), and *Salmonella* Typhimurium (ST19; Table 2).

**Table 2:** Various *Salmonella* serovars were identified from fecal samples of cattle in this study.

**Farm ID**	**Isolate ID**	**Serovar**	**Identified SNP differences**	**Serogroup**	**O antigen**	**h1**	**h2**	**Sequence type (ST)**	**H_2_S negative phenotype**
C	S1_A	Braenderup	26	C1	6,7,14	e,h	e,n,z15	22	–
	S1_B	Hadar	32	C2C3	6,8	z10	e,n,x	33	–
D	S2	Braenderup	26	C1	6,7,14	e,h	e,n,z15	22	–
	S3	Thompson	23	C1	6,7,14	k	1,5	26	–
	S4	Thompson	25	C1	6,7,14	k	1,5	26	–
	S5	Enteritidis	44	D1D2	1,9,12	g,m	–	11	–
F	S6	Thompson	23	C1	6,7,14	k	1,5	26	–
	S7_A	Hadar	29	C2C3	6,8	z10	e,n,x	33	–
	S7_B	Thompson	24	C1	6,7,14	k	1,5	26	–
	S8_A	Thompson	25	C1	6,7,14	k	1,5	26	–
	S8_B	Typhimurium	29	B	1,4,[5],12	i	1,2	19	+
Q	S9	Braenderup	27	C1	6,7,14	e,h	e,n,z15	22	–
R	S10_A	Hadar	32	C2C3	6,8	z10	e,n,x	33	–
	S10_B	Bareilly	32	C1	6,7,14	y	1,5	464	–
	S11	Hadar	30	C2C3	6,8	z10	e,n,x	33	–

_A and _B indicate that the isolates were recovered from the fecal samples of the same animal.

**Figure 1:**
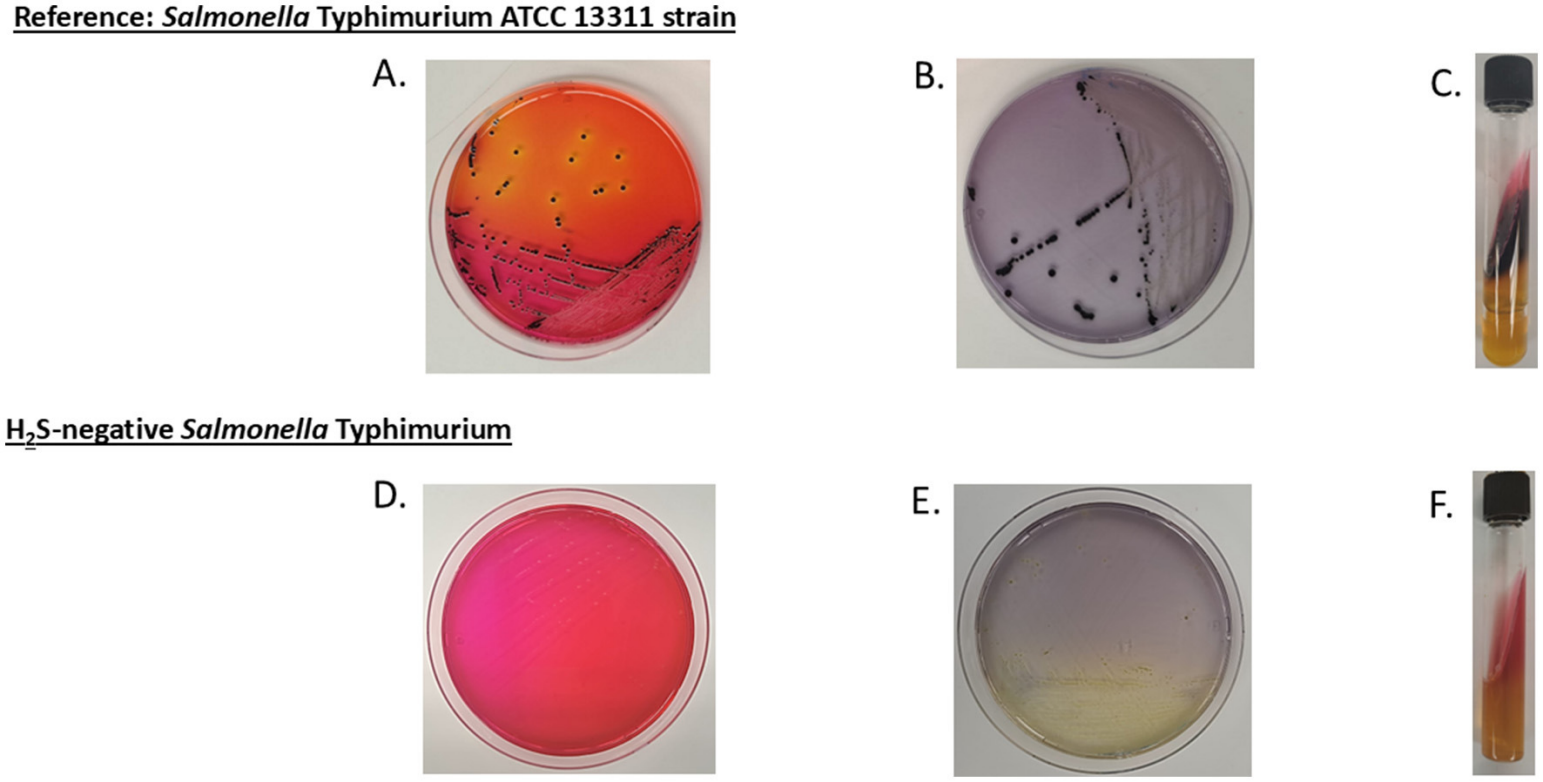
Colony morphology of reference and H_2_S-negative *Salmonella* on selective media. **(A–C)** display *Salmonella* Typhimurium ATCC 13311 (reference strain) cultured on xylose lysine deoxycholate (XLD) agar, lysine iron agar (LIA), and triple sugar iron (TSI) agar slant, respectively, showing the typical dark coloration morphology indicative of H_2_S production. **(D–F)** show the H_2_S-negative *Salmonella* Typhimurium isolate from this study cultured on the same respective media, exhibiting the absence of dark coloration due to the lack of H_2_S production.

Notably, four animals from three different farms were found to be co-shedding two distinct *Salmonella* serovars. The co-shedding combinations observed were as follows: Braenderup/Hadar, Thompson/Hadar, Bareilly/Hadar, and Thompson/Typhimurium (Table 2). The *Salmonella* Typhimurium isolate was identified as H_2_S-negative, based on its atypical appearance on XLD agar, LIA, and TSI agar (Figure 1).

### 3.2 Epidemiology of *Salmonella* on the sampled farms

Among the adult cattle sampled, 3.8% (nine out of 235) tested positive for *Salmonella*, compared to 2.6% (two out of 76) among calves (Table 3). All positive isolates were recovered from female cattle, representing 4% (11 out of 273) of the females tested. Based on fecal consistency, 6.6% (five out of 76) of diarrheic animals and 2.6% (six out of 235) of non-diarrheic animals tested positive for *Salmonella* (Table 3).

**Table 3:** Frequency of *Salmonella* detection among sampled cattle categorized by age, sex, and fecal consistency.

**Variable**	**Total No. sampled**	**Positives samples**	**Negatives samples**
		**No. (%)**	**No. (%)**
**Age**			
Calves	76	2 (2.6)	74 (97.4)
Adults	235	9 (3.8)	226 (96.2)
**Sex**			
Male	38	0 (0)	38 (100)
Female	273	11 (4)	262 (96)
**Diarrhea**			
Present	76	5 (6.6)	71 (93.4)
Absent	235	6 (2.6)	229 (97.5)

The results from the mixed-effects logistic regression analysis revealed considerable variation in the number of *Salmonella-*positive animals across farms. Diarrheic status was significantly associated with *Salmonella* shedding (*p*-value = 0.0192). However, neither the sex nor the age of the animals showed a statistically significant association with fecal shedding of *Salmonella* (Table 4).

**Table 4:** Summary statistics of a generalized mixed effects logistic regression analysis on samples.

		**Fixed effects.**		
**Intercept**	**Estimate**	**Std. error**	***z*** **-value**	***p*** **-value**
	−7.2656	2.9550	−2.459	0.0139^*^
Sex	−0.2048	1.3202	−0.155	0.8767
Age	1.4877	1.0105	1.472	0.1410
Diarrhea	2.7208	1.1619	2.342	0.0192^*^

* Indicates statistical significance; the farm groups were treated as a random effect, giving a variance and standard deviation of 10.06 and 3.171, respectively.

### 3.3 Antibiotic-resistance pattern of *Salmonella* isolates

**Figure 2:**
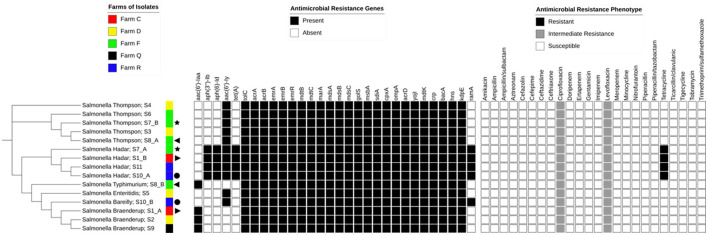
Antimicrobial resistance profiles and resistance genes among *Salmonella* isolates. A single-nucleotide polymorphism (SNP)-based phylogenetic tree is shown alongside the corresponding farm of origin, the antimicrobial resistance (AMR) genes, and phenotypic susceptibility profiles of the *Salmonella* isolates. Each isolate is labeled with its serovar name and isolate ID (as shown in Table 2), separated by a semicolon. The legends above each matrix provide an interpretation of the color-coded tiles, and the same symbols depict isolates from the same animal.

The major AMR genes and phenotypic resistance profiles identified among the *Salmonella* isolates are summarized in relation to the SNP-based phylogenetic tree (Figure 2). The number of SNP differences identified for each isolate, along with the isolate IDs used in the phylogeny, is presented in Table 2.

Notably, resistance was detected exclusively among the *Salmonella* Hadar isolates, all of which were resistant to tetracycline and harbored the *tet*(A) gene. Additionally, all isolates showed intermediate resistance to ciprofloxacin and levofloxacin. Detailed antimicrobial susceptibility profiles for each isolate are provided in the Supplementary material (Antimicrobial sensitivity).

The aminoglycoside resistance gene *aac(6*′*)-Iy* was detected in 11 out of 15 isolates. However, only four of the 15 isolates carried the *aph(3*″*)-Ib, aph(6)-Id*, and *aac(6*′*)-Iaa* genes (Figure 2).

### 3.4 Variation analysis of the H_2_S-negative isolate

**Figure 3:**
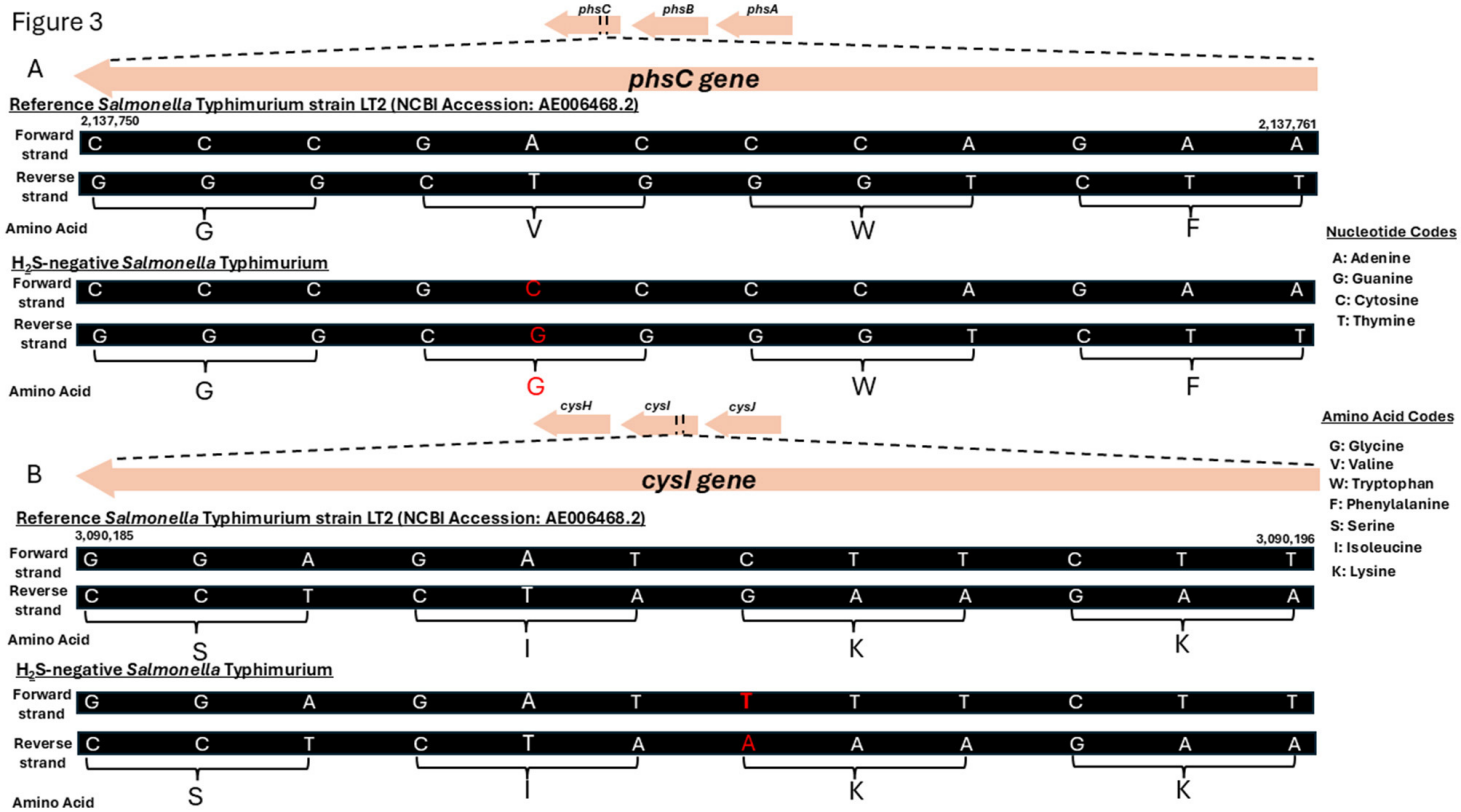
Genetic mutations identified in the *phsC* and *cysI* genes of the H_2_S-negative *Salmonella* Typhimurium isolate. **(A)** A schematic genome segment showing the *phsC gene* region (nucleotides 2,137,750 to 2,137,761) for both reference strain *Salmonella enterica* subsp. *enterica* serovar Typhimurium LT2 (NCBI Accession: AE006468.2) and the H_2_S-negative *Salmonella* Typhimurium isolate from this study. **(B)** A schematic genome segment showing the *cysI gene* region (nucleotides 3,090,185 to 3,090,196) in both the reference and the H_2_S-negative *Salmonella* Typhimurium isolate. Nucleotides and amino acids are shown in their single-letter codes. Mutated nucleotides and the corresponding amino acids (where a change in amino acids occurred) are highlighted in red.

Within the *phs* operon, a missense mutation (A > C) was identified in the *phsC* gene, resulting in an amino acid substitution from valine (V) to glycine (G) (Figure 3A). In the *cys* operon, a synonymous mutation (C > T) was detected in the *cysI* gene; however, this mutation did not lead to an amino acid change (Figure 3B). No genetic variations were observed in the *asr* operon. A complete list of the identified genetic variations and their predicted functional effects is provided in the Supplementary material (Variation analysis).

## 4 Discussion

This cross-sectional study targeted rural, small-scale cow–calf operations, a population often underrepresented in national-level surveillance due to geographic and demographic constraints. The most recent cow–calf study, organized by the National Animal Health Monitoring System (NAHMS) in the U.S., took place in 2017 (51). That survey reported a *Salmonella* prevalence of 1.0% at the herd level and 4.4% at the animal level among cow–calf populations (51). Alabama, one of the major cow–calf states included in the NAHMS survey, recorded 20,004 beef operations as of December 2017 (51).

Although our study does not match the scale of national surveillance efforts, the *Salmonella* prevalence we observed, 27.8% at the herd level and 3.5% at the animal level, is notably high, given our sample size of 311 animals. This discrepancy may arise from methodological differences, some of which are discussed below.

National studies typically rely on stratified sampling strategies designed to generate broad, generalizable inferences across states (52). In contrast, our investigation focused on multiple herds within a defined subregion of a single state, specifically targeting rural, small-scale cow–calf operations. Additionally, inherent sampling biases arising from farm selection criteria and reliance on owner consent were not controlled in this study. National-level surveillance efforts often offer incentives and resources that help reduce the impact of such limitations.

Moreover, findings from localized studies may reflect regional and time-dependent factors, as noted in earlier research (53). For example, cattle in the southern U.S. have been shown to shed *Salmonella* in feces at higher rates than those in the northern regions (54). Seasonal variation may also influence shedding; previous studies have reported increased *Salmonella* prevalence in dairy herds during the summer months (55). Since our study was conducted as a one-time, cross-sectional survey in the spring, future studies conducted in other seasons could provide valuable insights into seasonal trends in *Salmonella* shedding among these populations.

Additional confounding factors likely contributed to the observed prevalence. Notably, poor biosecurity practices were evident across several farms. For instance, none of the operations required visitors to wear boot covers, a basic yet critical preventive measure. Based on these observations, we recommend increased outreach through veterinary extension services and biosecurity training programs targeted at rural cattle producers. Such efforts could substantially reduce *Salmonella* transmission risk.

Finally, public education regarding the importance of research participation is essential in these communities. Engaging rural farmers in ongoing disease surveillance through community-centered outreach can enhance participation and enable more comprehensive studies that better capture the burden of disease among underrepresented cattle populations.

The higher frequency of *Salmonella* shedding observed among adult cattle (3.8%) compared to calves (2.6%) in our study was not statistically significant. Although sampling bias related to animal age may have influenced this outcome, a similar study investigating clinical salmonellosis in dairy cattle reported a markedly higher prevalence rate of 49.3% in adults and 23.8% in calves (56). In the same study, the prevalence of fecal *Salmonella* shedding in apparently healthy cattle was 10.1% for adults and 5.4% for calves (56). This age-related dynamic in *Salmonella* shedding may be partly attributed to the passive immunity that calves acquire through colostrum, which offers temporary protection against infection (57, 58). In our study, calves were not classified as either pre-weaned or weaned, limiting our ability to assess this effect more precisely. Future research that specifically distinguishes between pre-weaned and weaned calves would provide a more comprehensive understanding of *Salmonella* shedding dynamics in this age group.

Nevertheless, the high frequency of *Salmonella* shedding among adult cattle may be associated with waning immunity, underlying health conditions, or physiological stressors such as pregnancy. Additionally, adult cattle outnumbered calves in all the herds visited, resulting in a smaller calf sample size. This sampling imbalance may also have contributed to the apparent disparity in *Salmonella* shedding between age groups.

Our findings contrast with those reported by Cummings et al. (59), who observed a higher prevalence of *Salmonella* in calves compared to adult cattle. A potential explanation for this discrepancy is that all of the animals in their study were clinically ill and admitted to a veterinary hospital, a setting that may have influenced infection rates. The role of age as a risk factor for *Salmonella* infection in cattle remains inconclusive. In a previous study, a recent investigation also found no significant association between age and *Salmonella* shedding (60).

Interestingly, with regard to antimicrobial resistance, two of the four tetracycline-resistant isolates in our study were recovered from calves. Previous studies have shown that calves are more likely to harbor resistant *Escherichia coli* strains than adults (61, 62). This pattern has been consistently reported over decades across diverse geographic regions and farming practices (61). One explanation offered is the unique composition of the intestinal microbiome in young calves, which may favor the persistence of multidrug-resistant strains (61). While the presence of tetracycline-resistant *Salmonella* in calves, as observed in our study supports this hypothesis, further research is needed to substantiate this relationship, especially regarding *Salmonella* isolates from cattle.

Similar to age, the sex of cattle was not significantly associated with *Salmonella* shedding. However, just as there were more adults than calves in our sampled herds, there were also more females than males, which likely introduced sampling bias. These limitations undoubtedly influenced our findings. Therefore, the lack of significant associations between age or sex and *Salmonella* shedding in cow–calf operations in this study should be interpreted cautiously, taking these methodological constraints into account.

The association between diarrhea and fecal shedding of *Salmonella* was also investigated in this study. It is important to emphasize that the diarrheic state of animals cannot be conclusively attributed to either nutritional or infectious causes, as both factors can influence fecal consistency in cattle. However, all animals sampled appeared clinically healthy, and most farms maintained their herds on grass pastures, supplementing with Bermuda grass hay and soy hull pellets twice daily, along with *ad libitum* access to water. Sampling occurred at various times of the day, including before, during, and after feeding.

Despite these variables, fecal shedding of *Salmonella* among diarrheic animals was the only statistically significant parameter in our study. This finding aligns with earlier studies that have linked bovine enteritis to *Salmonella* shedding, a common clinical manifestation of salmonellosis in adult cattle (63). Similar associations have been documented in other research (64, 65). Notably, some non-diarrheic animals in our study were also found to be shedding *Salmonella*, supporting the notion that asymptomatic carriers can serve as a persistent source of infection (54). These subclinical shedders pose a risk of undetected transmission, emphasizing the importance of maintaining strict hygiene protocols for farmworkers handling both symptomatic and asymptomatic animals.

Our study also demonstrated that individual animals can shed multiple *Salmonella* serovars simultaneously, a phenomenon supported by previous research (66). This underscores the importance of selecting more than one presumptive colony during *Salmonella* isolation, when resources allow. However, this recommendation must be balanced with practical limitations, as dominant serovars may be repeatedly isolated due to the often indistinguishable colony morphology on selective agar media. The number of colonies selected for further analysis should therefore be guided by the researcher's discretion, resource availability, and specific study objectives.

A noteworthy observation from our study was the prevalence of *Salmonella* Thompson, *Salmonella* Hadar, and *Salmonella* Braenderup—serovars not commonly associated with cattle globally (67). Nonetheless, *Salmonella* Thompson and *Salmonella* Braenderup have been isolated from bovine lymph nodes (68), which are significant reservoirs of *Salmonella* and may contribute to human infection (69), particularly when these infected lymph nodes are inadvertently incorporated into ground beef (68). Recent studies have shown that a considerable proportion of human salmonellosis cases linked to beef are associated with ground beef products (70).

In addition to direct transmission through meat, these serovars have also been implicated in environmental contamination, leading to outbreaks. For example, a recent multistate outbreak of *Salmonella* Braenderup in the U.S. affected 551 individuals across 34 states (71–73). Traceback investigations and comparative genomic analyses linked the outbreak strain to a water source used on a vegetable farm (71). These findings highlight the potential for *Salmonella*-infected cattle feces to contaminate nearby water bodies through runoff, posing broader public health risks. Research suggests that reintroduction events by host animals may contribute to the persistent contamination of farm water systems (74).

As expected, the presence of the *tet(A)* gene in *Salmonella* Hadar isolates conferred phenotypic resistance to tetracycline. This finding is consistent with previous studies that identified *tet(A)* as the most prevalent tetracycline resistance gene in *Salmonella* (75). Interestingly, although aminoglycoside resistance genes such as *aph(3*″*)-Ib, aph(6)-Id, aac(6*′*)-Iy*, and *aac(6*′*)-Iaa* were detected in several isolates, none exhibited phenotypic resistance to aminoglycosides. This discrepancy aligns with earlier research in which resistance genes were identified in 37 *Salmonella* isolates without corresponding phenotypic resistance (76). These findings suggest that resistance genes may be functionally inactive or “silenced” due to regulatory mutations, disrupted expression pathways, or the presence of cryptic or non-functional elements (77, 78). In particular, the *aac (6*′*)-Iaa* gene has been previously reported as a cryptic gene (79).

A contrasting phenomenon was observed with ciprofloxacin and levofloxacin. Despite the absence of known quinolone resistance genes, such as *qnr, qep*, or *aac(6*′*)-Ib-cr* (80), and no impactful point mutations detected, all isolates exhibited intermediate resistance to these fluoroquinolones. Resistance to quinolones is multifaceted and is commonly associated with mutations in genes encoding DNA gyrase (*gyrA* and *gyrB*) and topoisomerase IV (*parC* and *parE*) (80). In our study, however, the intermediate resistance observed may instead be attributed to the activity of multiple efflux pumps, particularly those belonging to the SOS-box family, including *mdtABC-tolC, emrAB-tolC*, and *acrAB-tolC* (81). The *emrA* and *emrB* genes specifically encode efflux systems capable of expelling fluoroquinolones, thereby contributing to reduced susceptibility (81). Although some resistance patterns were shared among serovars isolated from different farms, further studies are needed to understand the dynamics of antimicrobial resistance and selection in small-scale farming systems.

To the best of our knowledge, this study is the first to report the isolation of an H_2_S-negative *Salmonella* Typhimurium strain from cattle feces. Globally, H_2_S-negative *Salmonella* Typhimurium isolates have been reported from various animal and human sources. For instance, these isolates have been recovered from retail chicken and pork products in Japan and China (8, 18), while a national surveillance effort in China identified H_2_S-negative *Salmonella* Typhimurium in humans and an unspecified livestock source (7). More recently, the monophasic variant of this serovar exhibiting the H_2_S-negative phenotype was isolated from raw chicken samples in Portugal (9), and in Sweden, the monophasic variant was implicated in a human salmonellosis outbreak (16). However, none of these reports documented the isolation of this atypical serovar from cattle feces. In the U.S., H_2_S-negative *Salmonella* has previously been detected in cattle, but the isolate belonged to the *Salmonella* Cerro serovar (20). The rarity of these atypical isolates likely reflects their ability to evade detection by conventional culture methods, although emerging evidence suggests that they are more widespread than previously assumed.

In terms of genetic mechanisms, the synonymous mutation observed in the *cysI* gene of our isolate is unlikely to have functional consequences, as such mutations are generally considered neutral (82). However, the missense mutation in the *phsC* gene may have functional implications, as such changes can affect protein stability and protein-protein interactions at the cellular level (83). Of the operons involved in H_2_S production in *Salmonella*, the *phs* operon, which includes the *phsA, phsB*, and *phsC genes*, is considered the most critical (14). Mutations in this operon, particularly in the *phsA*, have been reported to result in premature stop codons (9, 14), which truncates the thiosulfate reductase enzyme required for H_2_S production (12). Similar missense mutations in the *phs* and *cys* operons have also been described in previous studies (7, 14, 17). Nonetheless, it remains uncertain whether the *phsC* mutation identified in our isolate alone is sufficient to account for the H_2_S-negative phenotype observed. Further functional studies are needed to clarify the impact of these mutations on *Salmonella* metabolism and detectability.

We acknowledge that biases associated with our sampling strategy, particularly in relation to age group and sex, represent limitations of this study. These biases were largely due to the voluntary participation of farmers, which constrained our ability to randomize the study population. This challenge has also been noted in similar surveillance efforts (56). Despite these constraints, our study provides important insights into a largely underrepresented population of rural cattle farms and reports, for the first time, the isolation of an H_2_S-negative *Salmonella* Typhimurium serovar from cattle feces. Given the observed prevalence of *Salmonella* shedding, we recommend that extension services actively promote improved hygiene practices and biosecurity measures within cow–calf operations.

Furthermore, expanded studies involving larger sample sizes and broader geographic coverage are needed to provide a comprehensive overview of *Salmonella* prevalence and diversity in U.S. cattle and to better assess their public health implications. In conclusion, through an integrative approach combining classical microbiology, antimicrobial resistance profiling, and whole-genome sequencing, this study underscores the significant role that rural cow–calf herds may play as reservoirs for *Salmonella*.

## Data Availability

The datasets presented in this study can be found in online repositories. The names of the repository/repositories and accession number(s) can be found in the article/Supplementary material. Additional information can be found in the Supplementary materials. All raw sequence data generated in this study have been deposited in the National Center for Biotechnology Information (NCBI) under the BioProject accession number PRJNA1253333 and are accessible at: https://www.ncbi.nlm.nih.gov/bioproject/PRJNA1253333/.
